# Variation in Anti-inflammatory, Anti-arthritic, and Antimicrobial Activities of Different Extracts of Common Egyptian Seaweeds with an Emphasis on Their Phytochemical and Heavy Metal Contents

**DOI:** 10.1007/s12011-022-03297-1

**Published:** 2022-06-04

**Authors:** Aida H. Shobier, Mona M. Ismail, Sahar W. M. Hassan

**Affiliations:** National Institute of Oceanography and Fisheries (NIOF), Cairo, Egypt

**Keywords:** Seaweeds, Secondary metabolites, Anti-inflammatory activity, Antifungal activity, Biochemical content, Heavy metal composition

## Abstract

The anti-inflammatory, anti-arthritic, and antimicrobial activities of some common Egyptian seaweeds in addition to their phytochemical and heavy metal contents were investigated. Phytochemical screening of the seaweed extracts showed the presence of different primary and secondary metabolites with different concentrations according to their species and the used solvent. The ethanolic extract of *Colpmenia sinuosa* (CSBE2) exhibited the maximum anti-inflammatory and anti-arthritic activity at 1000 μg/ml concentration compared to other seaweed extracts. The dichloromethane extract of *Corallina officinalis* (CORM) exerted the highest antimicrobial activity with an average inhibition zone diameter (AV) = 15.29 mm and activity index (AI) = 1.53 and with the highest antagonistic activity against *Escherichia coli* (28 mm). It is followed by *Ulva linza* ethanolic extract (ULGE2) which recorded (AV) of 14.71 mm and (AI) of 1.30 with the highest antifungal activity against *Candida albicans* (30 mm). The collected seaweeds would therefore be a very promising source for treating inflammatory, arthritic, and microbial diseases. Moreover, the investigated seaweeds showed variable concentrations of heavy metals among various species. The mean concentrations of the heavy metals took the following order: Fe > Zn > Mn > Ba > Cu > As > Cr > Ni > Pb > V > Cd > Se > Co > Mo. Based on the permissible limits set by the WHO and CEVA, Pb and Ni in the studied seaweeds were found to be within the permissible limits, whereas Cd and Zn contents were at the borderline. Significant correlations were observed between studied parameters. The estimated daily intakes for most heavy metals were lower than the recommended daily intakes.

**Supplementary Information:**

The online version contains supplementary material available at 10.1007/s12011-022-03297-1.

## Introduction

The ocean is rich in various marine resources, comprising seaweeds. Among all biota living in the oceans, seaweeds have been known as valuable natural and renewable sources of bioactive compounds such as carbohydrates, proteins, lipids, phenolic compounds, polysaccharides, polyketides, alkaloids, phlorotannins, diterpenoids, sterols, halogenated compounds, and vitamins [1, 2]. These unique phytochemicals possess potent biological activities and diverse pharmacological potential like antimicrobial, anti-inflammatory, anti-arthritic, and antiproliferative activities [3, 4]. Seaweeds are also used for the development of new drugs [5, 6].

Inflammation is a complex process which is initiated by different factors such as chemical injury, bacterial infection, and environmental pollution [7]. Inflammation is a molecular indicator of carcinogenesis [8]. It has been reported that about 20% of human cancers are caused because of chronic inflammation [9]. Other diseases such as diabetes mellitus, obesity, and neurological diseases have been associated to inflammation [10].

Rheumatoid arthritis is an inflammatory autoimmune disease with chronic proliferative synovitis and synovial inflammation, as well as considerable bone and cartilage degradation, culminating in severe joint damage and limited functionality that requires long-term treatment with anti-inflammatory medicines [11]. Many inflammatory mediators, like tumor necrosis factor (TNF-), nitric oxide (NO), prostaglandins, reactive oxygen species (ROS), leukotrienes, and enzymes (lipoxygenases, cyclooxygenases (COX-1 and COX-2), and phospholipases), play a key role in bone destruction and synovial membrane inflammation during the development of rheumatoid arthritis [12]. It affects approximately 1% of the global population with a female to male ratio of 2.5:1 [13]. Recently, the development of new safe, potent, and less toxic anti-arthritic drugs is an increasing concern worldwide. As a result, new naturally available alternatives have been investigated in order to assess and expand their therapeutic potential.

Numerous marine natural compounds isolated from several seaweeds were reported to exhibit powerful anti-inflammatory activity [14]. For example, the sulfated polysaccharide fraction extracted from the green seaweed *U. lactuca* showed high anti-inflammatory activity at a low dose of 1 mg/kg [3]. The hydroethanolic extract of *U. lactuca* demonstrated anti-arthritic activity which may be due to its antioxidant and anti-inflammatory potential [3].

The resistance to antibiotics has become a challenge for the society and public health, causing reemerge of infectious diseases and subsequent risk to health of human. The resistance genes to antibiotics are exist within the surrounding environment and can be gained by pathogenic microorganisms via horizontal gene transfer. Novel antibiotics that are presented in the market face the increase of microbial resistant after a definite time of use [15].

Prospecting for novel antibiotics is a labor-rigorous work due to the presence of some obstacles such as low yields of the produced compounds. Consequently, the accessible ability for discovery should be concentrated on the most talented sources for bioactivity and chemical novelty using the suitable scientific tools [15].

Seaweeds have become goals for searching of novel compounds that exhibit prospective medical values, including antimicrobial activity [16]. Many researches are motivated on seaweeds crude extracts gathered from Mediterranean seashores due to their compelling antimicrobial activity [4, 17]. The produced chemical composition varies between seaweeds according to the environmental conditions, habitats, species, and maturity [18].

Essential metal micronutrients, such as Cu, Zn, Fe, Mn, Co, Cr, Se, Mo, and V, are required in small quantities to maintain the metabolism of the human body. But at higher concentrations, they can lead to poisoning [19]. Non-essential elements, such as Pb, Cd, As, and Hg, have no known biological role in organisms and exhibit their toxicity by competing with essential metals for the sites of active enzyme or membrane protein [20]. These non-essential elements are nonbiodegradable and do not possess any positive effects on organisms and are toxic at low doses [21]. They cannot be metabolized into nontoxic forms and accumulate in the human body to cause health problems [20].

Furthermore, the environmental impact of these pollutants on the marine ecosystem must be determined since these pollutants cause unfavorable changes in the biological and physicochemical factors of the aquatic ecosystem [21]. According to the World Health Organization (WHO), the levels of heavy metals must be controlled so that public safety has been achieved [22]. Consumption of seaweeds, grown in polluted areas, leads to harmful effects on human health [23]. In order to get good therapeutic benefits, quality of seaweeds products must be ascertained in terms of metal contamination.

The main objective of the present study was screening and comparing the ability of different solvent seaweed extracts (*Ulva fasciata*, *Ulva linza*, *Corallina officinalis*, *Jania rubens*, and *Colpomenia sinuosa*) gathered from the Alexandria coast, Egypt, for potential anti-inflammatory, anti-arthritic, and antagonistic effect against different bacterial and fungal pathogens for subsequent use in some therapeutic applications. Their phytochemical and heavy metal contents as well as the estimated daily intakes for some selected heavy metals (Fe, Mn, Zn, Cu, Co, Cr, Ni, Pb, Cd, V, As, Se, Mo, and Ba) have been also evaluated.

## Materials and Methods

### Collection of Seaweeds

Fresh seaweeds (*Ulva fasciata* Delile, *Ulva linza* Linnaeus, *Corallina officinalis* Linnaeus, *Jania rubens* (Linnaeus) Lamouroux, and *Colpomenia sinuosa* (Mertens Ex Roth) Derbes and Solier) were collected from the Egyptian Mediterranean coast of Alexandria, at Abu Qir Bay, and the Eastern Harbor during the winter of 2018. Seaweed identification was made according to Aleem [24]. The names of the species were confirmed according to Algae Base website [25]. After sample collection, they were washed with running water to remove any associated debris and epiphytes, then washed with distilled water, and dried under shade and in the oven at 45 °C for 24 h. The dried seaweeds were grinded into fine powder and stored until analysis.

### Preparation of Crude Seaweed Extracts

The pulverized sample of each seaweeds species (25 g) was immersed in *n*-hexane followed by dichloromethane then 70% ethanol at room temperature for 1 week with continuous shaking. The obtained extracts were filtered and evaporated under reduced pressure at 45 °C to yield hexane (UFGH1, UFGH2, ULGH, JRRH, and CSBH), dichloromethane (UFGM1, UFGM2, ULGM, CORM, JRRM, and CSBM), and ethanolic (UFGE1, UFGE2, ULGE1, CORE1, JRRE1, and CSBE1) extracts. The extraction process was repeated twice again. The ethanolic extracts (UFGE3, UFGE4, ULGE2, CORE2, JRRE2, and CSBE2) were obtained directly by soaking in 70% ethanol for 2 weeks. The produced crude extracts were stored at − 20 °C until used for biological screening [4, 17].

### Qualitative Analysis of Phytochemical Content

Nine qualitative analyses were carried out for phytochemical screening of different crude extracts of the tested seaweeds. In these analyses, general reactions revealed the presence or absence of these compounds (terpenes, saponins, tannins, phlobatannins, steroids, flavonoids, coumarins, quinones, and cardiac glycosides) in the investigated extracts according to standard procedures [26, 27].

### Quantitative Analysis of Biochemical Content

Biochemical analysis including total protein content was determined according to the method of Lowry [28]. Carbohydrate content was measured following the phenol sulfuric acid method [29] and compared with glucose as a reference sugar. The amount of total phenol in different extracts was determined with Folin-Ciocalteu reagent according to the method of Kim et al. [30] with gallic acid as standard. Tannin content of various extracts was measured using Folin-Ciocalteu reagent assay according to Tambe and Bhambar [31].

### Anti-inflammatory Activity (15-lipoxygenase Inhibitory Assay)

Anti-lipoxygenase activity assay was done followed Pinto et al. [32] method with minor modifications. The assay was based on measuring the formation of the complex Fe^3+^/xylenol orange using a spectrophotometer at 560 nm. The percentage inhibition was calculated using Eq. (1). Quercetin was used as a standard drug.

1 $$\mathrm{\%\;inhibition}=\frac{[\left(A\mathrm{\;control}-A\mathrm{\;blank}\right)-(A\mathrm{\;sample}-A\mathrm{\;blank})] }{(A\mathrm{\;control}-A\mathrm{\;blank}) }\mathbf{*}100$$

### Anti-arthritic Activity (Protein Denaturation Assay)

Anti-denaturation activity of the tested extracts was done by Sakat et al. [33] method with slight modifications. Diclofenac sodium and distilled water were used as positive and negative controls, respectively. Percentage inhibition was measured at 660 nm and calculated using the following formula Eq. (2): The experiment was carried out in triplicate. Diclofenac sodium was used as a standard drug.

2 $$\mathrm{\%\;inhibition}=\frac{A\mathrm{\;sample}-A\mathrm{\;blank}}{A\mathrm{\;control}}*100$$

where *A* control is the absorbance of control, *A* blank is the absorbance of blank, and *A* sample is the absorbance of the sample.

### Test Pathogenic Microorganisms

The indicator pathogenic microorganisms including pathogenic bacteria (*Staphylococcus aureus*, *Escherichia coli*, *Pseudomonas aeruginosa*, *Enterococcus faecium*, *Klebsiella pneumoniae*) and pathogenic fungi (*Candida albicans* and *Fusarium solani*) were kindly obtained from the Marine Microbiology Lab, Division of Marine Environment, National Institute of Oceanography and Fisheries (NIOF).

### Antimicrobial Assay

Antimicrobial activity of the tested seaweed extracts against the selected pathogens was carried out using well-cut diffusion technique [34], followed by incubation at 37 °C for 24 h. Positive results were detected as a clear zone around each well and measured in mm. Fusidic acid (5 µg) was used as a positive control and DMSO was considered as a negative control.

### Activity Index

The activity index (AI) was used for comparing the antimicrobial activity of each tested seaweed extract against all pathogens with that obtained from standard antibiotic Eq. (3).

3 $$\mathrm{AI}= \frac{\mathrm{Mean\;of\;the\;extract\;inhibition\;zone\;diameter}}{\mathrm{Mean\;of\;the\;standard\;antibiotic\;drug\;inhibition\;zone\;diameter}}$$

### Elemental Analysis

**Fig. 1:**
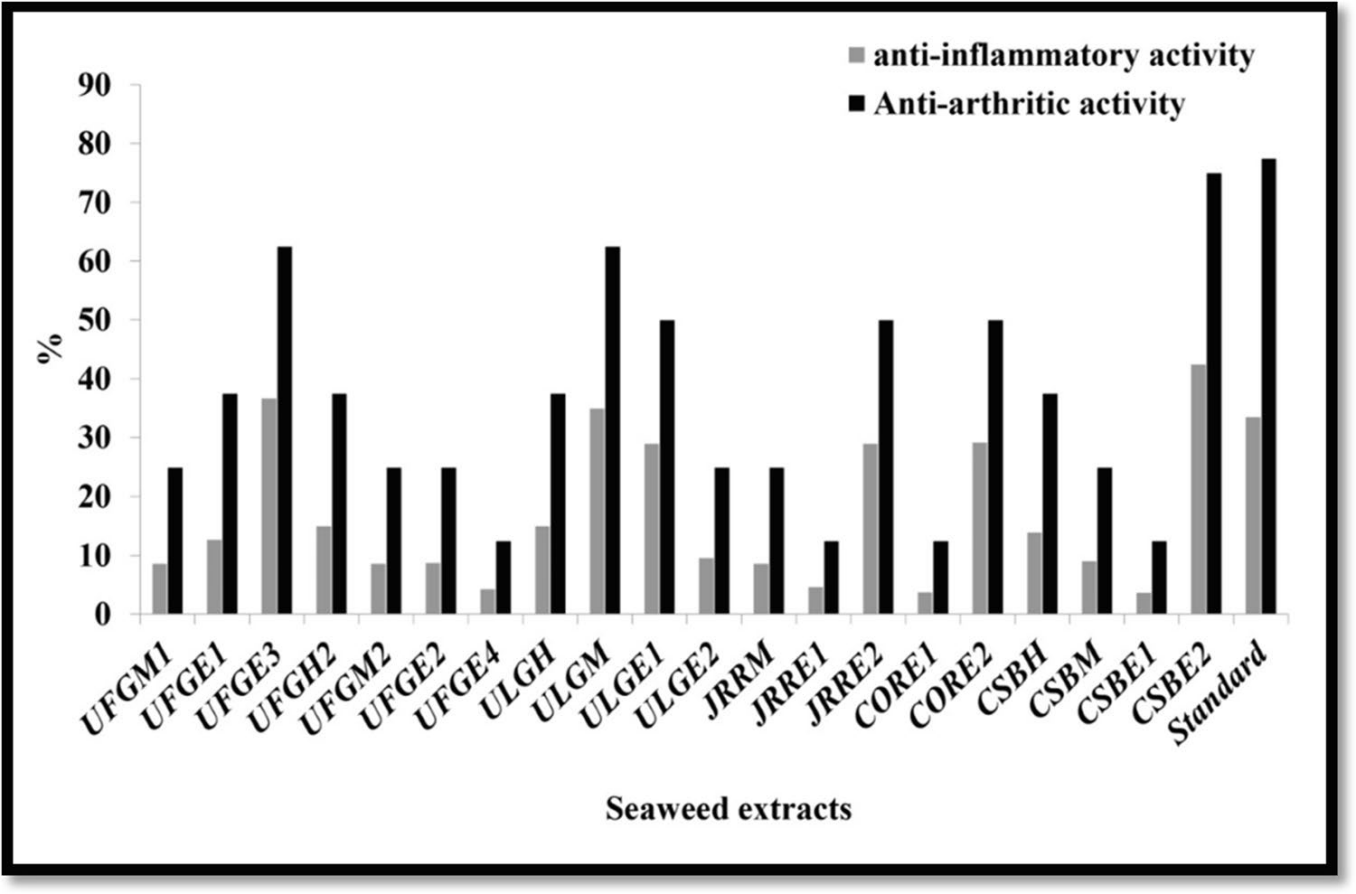
In vitro anti-inflammatory and anti-arthritic activities % of the selected seaweed extracts

0.5 g of each powdered seaweed sample was digested with 5 ml of concentrated HNO_3_ (Merck, Germany) inside stoppered Teflon vessels at 70–80 °C. After cooling, 2 ml of 30% H_2_O_2_ was added then heated until complete digestion [35]. After digestion, the samples were diluted to 25 ml with deionized water and then filtered by Whatman No. 1. Heavy metals concentrations were measured by inductively coupled plasma optical emission spectroscopy (ICP-OES, Agilent’s 5100 VDV). Analytical calibration curves of the analyzed metals are shown in Fig. (1S).

### Estimated Daily Intake (EDI)

The estimated daily intake for the investigated elements (Fe, Mn, Zn, Cu, Co, Cr, Ni, Pb, Cd, V, As, Se, Mo, and Ba) in their noncancerous health contents was determined using Eq. (4): [36].

4 $$\mathrm{Estimated\;daily\;intake\;}\left(\mathrm{mg}/\mathrm{kg}/\mathrm{day}\right)= \frac{C\times IR \times EF \times ED}{BW \times AT}$$

where *C* is the average concentration (in milligrams per kilogram) of the contaminant; *IR* is the ingestion rate (0.227 kg/day (8-oz. meal) for adult); *EF* is the exposure frequency (365 days/year); *ED* is the exposure duration (70 years); *BW* is the body weight (70 kg); and *AT* is the averaging time (noncancer/lifetime = ED × 365 days/year).

### Statistical Analysis

Correlation matrix (*r*) was carried out using the STATISTICA 99 software, version 12.0 at a significance level of *p* ≤ 0.05 in order to find out the relationships between different estimated parameters.

## Results and Discussion

### Phytochemical Composition

The phytochemical analysis of different crude seaweed extracts (Table 1) showed the presence of valuable secondary metabolites such as terpenes, tannins, steroids, flavonoids, and coumarins in nearly all tested extracts. Meanwhile, saponins, quinones, and cardiac glycosides were rarely detected. The various phytoconstituents were different according to seaweeds species, their chemical composition, the used solvents, habitat, time of collection, and physico-chemical parameters of water [37].

**Table 1:** Preliminary phytochemical screening of different extracts of the tested seaweeds

Seaweed species	Extract	Code	Terpenes	Saponins	Tannins	Phlobatannins	Steroids	Flavonoids	Coumarins	Quinones	Cardiac glycosides
*U. fasciata (EH)***)**	Dichloromethane	UFGM1	*	**-**	*	**-**	-	*	**-**	**-**	**-**
	Ethanolic	UFGE1	*	*	*	*	*	-	**-**	**-**	**-**
		UFGE3	*	*	*	**-**	-	*	*	**-**	**-**
*U. fasciata (AQ)*	Hexane	UFGH2	**-**	**-**	*	**-**	-	-	**-**	**-**	**-**
	Dichloromethane	UFGM2	*	*	*	*	*	-	*	**-**	**-**
	Ethanolic	UFGE2	*	**-**	*	*	-	**-**	*	**-**	**-**
		UFGE4	*	**-**	*	**-**	*	-	**-**	*	**-**
*U. linza (AQ)*	Hexane	ULGH	**-**	**-**	*	**-**	*	*	**-**	**-**	**-**
	Dichloromethane	ULGM	*	**-**	*	**-**	*	*	*	**-**	**-**
	Ethanolic	ULGE1	*	**-**	*	*	*	-	**-**	**-**	**-**
		ULGE2	*	**-**	*	*	-	-	**-**	**-**	**-**
*J. rubens (AQ)*	Dichloromethane	JRRM	*	*	*	**-**	*	*	*	**-**	**-**
	Ethanolic	JRRE1	*	**-**	*	**-**	-	-	**-**	**-**	**-**
		JRRE2	*	**-**	*	**-**	*	*	**-**	*	**-**
*C. officinalis (EH)*	Ethanolic	CORE1	*	**-**	*	*	-	*	*	*	**-**
		CORE2	*	**-**	*	**-**	*	*	**-**	**-**	**-**
*C. sinuosa (AQ)*	Hexane	CSBH	**-**	**-**	*	**-**	**-**	*	**-**	*	**-**
	Dichloromethane	CSBM	*	**-**	*	**-**	*	*	**-**	*	**-**
	Ethanolic	CSBE1	*	**-**	*	*	*	*	*	*	**-**
		CSBE2	*	*	*	*	*	*	*	**-**	**-**

^*^Present; -Absent

These compounds are of considerable pharmaceutical importance since they are used as many drugs. For instance, terpenes and steroids from marine algae are the classes of anti-inflammatory compounds found ubiquitously. Terpenoids have anti-inflammatory and hypoglycemic activities [38]. Saponins showed antimicrobial, anti-inflammatory, and hemolytic effects [39]. Tannins exhibited inflammatory effects which control agitation in the small intestine and all indications of gastritis, esophagitis, enteritis, and irritating bowel disorders [40]. Cardiac glycosides are the main ingredient in heart drugs for treatment of cardiac failure and atrial arrhythmias [41].

### Biochemical Composition

The qualitative analyses of biochemical content revealed the presence of carbohydrates, proteins, phenols, and tannins in all selected seaweed extracts (Table 2). Carbohydrate content ranged between 8.97 and 534.65 mg/g dw in dichloromethane extract (UFGM1) and ethanolic extract (UFGE3) of *U. fasciata,* respectively. This variation is related to the difference in the solubility of the algal chemical content with the used solvents [42].

**Table 2:** Biochemical composition of different extracts of the tested seaweeds

Seaweed species	Extract	Code	Carbohydrates (mg/g)	Proteins (mg/g)	Phenols (mg/g)	Tannins (mg/g)
*U. fasciata (EH)*)	Dichloromethane	UFGM1	8.97	0.02	0.43	0.05
	Ethanolic	UFGE1	16.37	8.69	8.89	1.67
		UFGE3	534.65	22.67	21.66	4.10
*U. fasciata (AQ)*	Hexane	UFGH2	10.53	0.01	0.07	0.09
	Dichloromethane	UFGM2	51.80	1.66	0.66	0.27
	Ethanolic	UFGE2	99.14	1.63	7.20	0.84
		UFGE4	429.84	5.22	12.86	2.32
*U. linza (AQ)*	Hexane	ULGH	34.79	0.01	2.89	0.26
	Dichloromethane	ULGM	75.68	1.32	12.19	0.40
	Ethanolic	ULGE1	398.98	9.36	8.35	1.04
		ULGE2	122.96	19.52	15.36	3.64
*J. rubens (AQ)*	Dichloromethane	JRRM	66.26	0.03	1.90	0.25
	Ethanolic	JRRE1	34.69	2.21	2.81	0.45
		JRRE2	166.22	7.03	29.07	1.11
*C. officinalis (EH)*	Ethanolic	CORE1	164.10	6.68	3.16	0.76
		CORE2	26.29	7.03	6.30	0.92
*C. sinuosa (AQ)*	Hexane	CSBH	18.30	0.02	0.01	0.14
	Dichloromethane	CSBM	46.63	1.59	1.35	3.42
	Ethanolic	CSBE1	39.46	1.77	0.00	3.17
		CSBE2	203.04	12.41	29.26	4.55

The protein content of the selected seaweeds varied from 0.01 mg/g dw in hexane extracts of *U. fasciata* (UFGH2) and *U. linza* (ULGH) to 22.67 mg/g dw in *U. fasciata* ethanolic extract (UFGE3). These values were similar to the obtained results by Ismail et al. [43]. The difference in protein content may be attributed to differences in the seaweed species and the used solvent. The hexane extracts of *U. fasciata* (UFGH2), *U. linza* (ULGH), and *C. sinuosa* (CSBH) as well as the dichloromethane extracts of *U. fasciata* (UFGM1) and *J. rubens* (JRRM) were characterized by trace concentration of protein content ~ 0.02 mg/g dw which may be due to the polarity of the used solvents and seaweed species. The protein content of seaweeds is dependent mainly on the season and the environmental conditions [44].

In the present study, the polyphenolic compounds (phenols and tannins) were different between seaweed species and solvents. Higher phenol content was found in the ethanolic extract of *C. sinuosa* (CSBE2; 29.26 mg/g dw) and lower in hexane extract of the same alga (CSBH; 0.01 mg/g dw). The maximum tannin content (4.55 mg/g dw) was recorded in CSBE2, while the dichloromethane extracts of *U. fasciata* (UFGM1) recorded the minimum content (0.05 mg/g dw). Brown seaweeds contain higher amounts of polyphenols than green seaweeds [45].

Hassan and Shobier [17] demonstrated the chemical compositions of the ethanolic extract of *C. sinuosa* and found that the most abundant components were tridecanoic acid ethyl ester, tetradecanoic acid, ethyl ester, *n*-hexadecanoic acid, ethyl (9*Z*,11*E*)-9,11-octadecadienoate, ethyl *n*-heptadecanoate, bis (2-ethylhexyl) 1,2-benzenedicarboxylate, 6,10,14-trimethylpentadecan-2-one, and *n*-pentadecanoic acid ethyl ester.

Of all the three solvent extracts, ethanolic extract of the seaweeds showed a maximum number of active components than hexane and dichloromethane extracts so ethanol was the best solvent to screen the bioactive compounds. This result conforms to the finding of Plaza et al. [46] who recommended ethanol as the most suitable solvent for extracting the bioactive compounds.

### Anti-inflammatory Activity

Lipoxygenase enzymes are one of the most potent natural mediators of hypersensitivity and inflammation which catalyze the conversion of arachidonic acid to hydroperoxy eicosatetraenoic acids (HPETEs), and then reduced to mono-hydroxy eicosatetraenoic acids (mono-HETEs) or (diHETEs) and leukotrienes; these are ranked among the most potent natural mediators of hypersensitivity and inflammation [47]. Figure 1 shows that all the seaweed extracts had various inhibitory activity against 15-lipoxygenase in response to seaweed species and phytochemical content of various extracts [48]. The anti-inflammatory effect of seaweeds is related to their active phytochemical contents such as tannins, flavonoids, polyphenols, alkaloids, curcamins, catechins, vitamins, and β-carotene which had the abilities to moderate cell mitogen-activated protein kinases (MAPK) signaling pathways, proliferation, apoptosis, and redox balance besides. So these compounds are protective agents against many diseases such as cancer, neurodegenerative disorders, and cardiovascular [49]. The anti-inflammatory of polyphenol compounds may be mediated by suppressing effect of necrosis factor “NF-kB” and AP-1 transcription [50].

The ethanolic extracts of *C. sinuosa* (CSBE2) and *U. fasciata* (UFGE3) as well as the dichloromethane extract of *U. linza* (ULGM) had the best anti-lipoxygenase activity with maximum percentage of inhibition of 42.5%, 36.37%, and 35.02% at a concentration of 1000 μg/ml, respectively more than the positive control quercetin (1000 µg/ml) which had 33.52%. Our results are similar to the observation of Souza et al. [49] who demonstrated the anti-inflammatory ability of the aqueous and methanolic extracts of *Caulerpa mexicana* and *Gracilaria changii* which may be attributed to their alkaloid content having an indirect effect on inflammatory mechanisms.

### Anti-arthritic Activity

The ethanolic extract of *C. sinuosa* (CSBE2 showed a maximum anti-denaturation activity (75%) followed by the ethanolic extract of *U. fasciata* (UFGE3; 62.5%) and the dichloromethane extract of *U. linza* (ULGM; 62.5%) (Fig. 1).These extracts exhibited their ability to control the production of autoantigens and thereby they inhibit the denaturation of proteins compared with the drug diclofenac sodium where the major cause of rheumatoid arthritis is denaturation of proteins and production of autoantigens [51]. This ability may be related to the presence of secondary metabolites like steroids, alkaloids, and flavonoids [52]. Our results were higher than those reported by Sumanya et al. [53] who reported that the maximum anti-arthritic activity of *Caulerpa racemosa* methanolic extract was 49.33 ± 0.60%.

### Antimicrobial Activity

It was observed that the tested seaweed extracts exhibited varied antimicrobial activity against the selected pathogens (Table 3). The antibacterial and antifungal activities of seaweeds were reported in several previous studies [4, 54, 55]. The highest antimicrobial activity was recorded for dichloromethane extract of *C. officinalis* (CORM) with an inhibition zone diameter ranged from 12 to 28 mm, recording an average inhibition zone diameter of 15.29 mm and an activity index of 1.53. It was observed that *E.coli* was the most sensitive pathogen for the bioactive metabolites in the crude extract. Hassan and Shobier [17] reported that the dichloromethane extract of *C. officinalis* contains several components that have been reported to possess antimicrobial activity such as *n*-nonadecane, 1-docosene, 1,2,3-propanetricarboxylic acid, 2-(acetyloxy)-tributyl ester, 1,1-dimethyltetradecyl hydrosulfide, 1-eicosanol, (17*E*)-17-pentatriacontene, and 2-octadecoxyethanol.

**Table 3:** Antimicrobial activity of the selected seaweed extracts against different pathogens

Seaweed species	Extract	Code	Inhibition zone diameter (mm)								AI
			*S. aureus*	*E. coli*	*P. aeruginosa*	*E. faecium*	*K. pneumoniae*	*C. albicans*	*F. solani*	AVG	
*U. fasciata (EH)*	Hexane	UFGH1	0	0	0	11	0	0	12	3.29	0.29
	Dichloromethane	UFGM1	0	7	0	0	6	0	0	1.90	0.17
	Ethanolic	UFGE1	15	15	14	15	15	0	0	10.57	0.94
		UFGE3	15	20	0	0	11	17	0	9.00	0.80
*U. fasciata (AQ)*	Dichloromethane	UFGM2	12	0	0	0	0	0	0	1.70	0.15
	Ethanolic	UFGE2	0	0	10	7	0	0	0	2.43	0.22
		UFGE4	12	10	12	0	12	15	0	8.71	0.77
*U. linza (AQ)*	Dichloromethane	ULGM	0	0	0	0	11	0	0	1.57	0.14
	Ethanolic	ULGE1	0	0	0	10	10	0	0	2.90	0.26
		ULGE2	15	15	15	15	13	30	0	14.71	1.30
*J. rubens (AQ)*	Hexane	JRRH	0	0	0	0	0	0	12	1.70	0.15
	Dichloromethane	JRRM	12	0	0	0	0	12	0	3.42	0.30
	Ethanolic	JRRE1	10	10	0	10	11	0	0	5.86	0.52
		JRRE2	15	20	0	0	0	15	0	7.14	0.63
*C. officinalis (EH)*	Dichloromethane	CORM	20	28	12	14	15	18	0	15.29	1.53
	Ethanolic	CORE1	10	10	12	13	13	10	0	9.70	0.86
		CORE2	0	0	0	0	0	0	12	1.70	0.15
*C. sinuosa (AQ)*	Hexane	CSBH	0	0	0	0	0	0	15	2.14	0.19
	Dichloromethane	CSBM	13	0	12	0	0	0	0	3.57	0.32
	Ethanolic	CSBE1	20	19	0	0	0	20	0	8.43	0.75
		CSBE2	20	15	15	16	16	20	0	14.57	1.29
Standard antibiotic	Fusidic acid		17	0	15	15	17	15	0	11.29	

*AVG*, average of inhibition zone; *AI*, activity index

Erturk and Tas [56] investigated the antibacterial and antifungal activity of seven seaweeds belonging to Chlorophyta, Phaeophyta, and Rhodophyta against the pathogenic bacteria (*E. coli*, *Listeria monocytogenesis*, *S. aureus*, *P. aeruginosa*, *Bacillus cereus*, and *Salmonella typhimurium*) in addition to two fungus (*Aspergillus niger* and *C. albicans*). In accordance with our study, it was reported that the extracted bioactive compounds from *C. officinalis* exhibited an antimicrobial activity against all tested pathogens recording the highest antibacterial activity against *E. coli* and showing an inhibition zone diameter of 15 mm, which seems to be lower than the antibacterial effect against *E. coli* realized in the current investigation (28 mm).

The ethanolic extracts showed reasonable antimicrobial activity against the tested pathogens symbolized as the ethanolic extract of *U*. *linza* (ULGE2) with a range of inhibition zone of 13–30 mm, average inhibition zone diameter of 14.71 mm, and activity index 1.30. Similarly, Barzkar et al. [57] stated that extracts of *U. fasciata* collected from India exhibited antibacterial activity with a broad spectrum against *E. coli*, *B. subtilis*, and *Aeromonas hydrophila*. Moreover, Erturk and Tas [56] reported that the ethanolic extract of *Ulva* sp. isolated from the coast of Vona Bay (Turkey) displayed antibacterial activity against *E. coli*, *P. aeruginosa*, *S. aureus*, and *B. subtilis* with inhibition zone diameter ranged between 10 and 15 mm. On the other hand, the methanolic:toluene extract of *U. fasciata* inhibited 40% of the tested bacteria [58].

This was followed by the ethanolic extract of *C. sinuosa* (CSBE2) which exhibited inhibition zone diameter of 15–20 mm, average zone diameter of 14.57 mm, and activity index 1.29 with the highest antagonistic effect against *S. aureus *and* C. albicans*. Mhadhebi et al. [59] reported the antifungal activity of different seaweeds against *C. albicans* with inhibition zone diameter ranged from 9 to 25 mm. Also, Ertürk and Tas [56] stated that extracts of *U. rigida* showed activity against *C. albicans* (12 mm). Lower average inhibition activity was detected for other solvent extracts showing the lowest activity for hexane extract of *J.* rubens (JRRH), ethanolic extract of *C. officinalis* (CORE2), and dichloromethane extract of *U. fasciata* (UFGM2) recording equal average inhibition zone of 1.7 and 0.15 activity index.

Overall results indicated that ethanol and dichloromethane solvents were more efficient for extracting the active metabolites. Bansemir et al. [60] studied the antibacterial activity of the metabolites extracted from 26 seaweed species using solvents with different polarity and reported that the highest activities were noted for the metabolites extracted by dichloromethane due to the hydrophobic nature of some constituents [61]. It was mentioned that the antimicrobial activity could be related to the combination of compounds not to a single compound and the difference in the antimicrobial activity may be attributed to natural factors such as pollution and environmental conditions. The time of sample collection, the reproductive state, and age of the seaweeds are other effective factors [17].

### Heavy Metal Content

The concentrations of fourteen heavy metals (Fe, Mn, Zn, Cu, Co, Cr, Ni, Pb, Cd, V, As, Se, Mo, and Ba) in the tested seaweed species from Abu Qir Bay and the Eastern Harbor have been evaluated. The mean concentrations of metals in the examined seaweeds decreased in the following order: Fe > Zn > Mn > Ba > Cu > As > Cr > Ni > Pb > V > Cd > Se > Co > Mo (Table 4).

**Table 4:** Concentrations of heavy metals in seaweeds collected from the Egyptian coast of Alexandria (mg/kg)

Seaweed species	Fe	Mn	Zn	Cu	Co	Cr	Ni	Pb	Cd	V	As	Se	Mo	Ba
Green seaweeds														
*U. fasciata (EH)*	146.70	24.55	40.62	16.62	0.25	2.62	3.80	2.57	0.46	0.58	3.35	0.03	0.07	6.07
*U. fasciata (AQ)*	402.90	17.70	43.30	16.83	0.20	8.02	5.51	0.89	0.10	2.71	6.60	0.05	0.25	7.70
*U. linza (AQ)*	899.96	21.11	93.47	19.27	0.30	7.49	4.43	1.15	0.32	3.49	9.06	0.03	0.20	12.35
Red seaweeds														
*J. rubens (AQ)*	414.99	85.36	30.99	5.04	0.26	2.01	2.10	3.33	3.65	1.73	2.10	0.54	0.28	11.92
*C. officinalis* (*EH)*	78.83	19.22	72.97	13.12	0.47	1.16	2.44	4.66	2.91	0.80	0.73	1.02	0.05	12.53
Brown seaweeds														
*C. sinuosa* (*AQ*)	572.02	50.62	24.38	5.34	0.30	2.63	2.53	2.98	1.52	3.13	4.79	1.09	0.30	34.93
Minimum	78.83	17.70	24.38	5.04	0.20	1.16	2.10	0.89	0.10	0.58	0.73	0.03	0.05	6.07
Maximum	899.96	85.36	93.47	19.27	0.47	8.02	5.51	4.66	3.65	3.49	9.06	1.09	0.30	34.93
Average	419.23	36.43	50.95	12.70	0.30	3.99	3.47	2.60	1.50	2.07	4.44	0.46	0.19	14.25
SD	298.29	26.90	26.69	6.14	0.09	2.97	1.34	1.41	1.49	1.22	3.05	0.50	0.11	10.48

Iron is one of the most important components of organisms such as algae and of enzymes like cytochromes and catalase, as well as of oxygen transporting proteins, like myoglobin and hemoglobin [62]. Excess iron uptake increases the risk of cancer since iron can initiate cancer by the oxidation of DNA molecules [63]. In this study, Fe level highly exceeded the permissible limit suggested by the FAO/WHO [64] for a medicinal plant (20 mg/kg) [65]. It recorded the highest concentration in the studied seaweeds and ranged in a wide interval (78.83 to 899.96 mg/kg for *C. officinalis* and *U. linza*, respectively). Fe concentration followed the order *U. linza* > *C. sinuosa* > *J. rubens* > *U. fasciata* (AQ) > *U. fasciata* (EH) > *C. officinalis*. The Indian algae had the mean iron content ranging between 3.70 and 6470 mg/kg as reported by Gopinath et al. [66]. The presence of high Fe concentration in marine plants may be attributed to its role in metabolic processes of the plant. Moreover, increased photosynthesis and respiration in marine plants allow them to uptake more Fe.

Manganese is an element possessing low toxicity and has considerable biological significance. It is considered one of the most biogeochemical and active transition metals in marine environment [67]. US Environmental protection Agency has mentioned that manganese is not classified as human carcinogen [68]. Mn concentration varied from 17.70 to 85.36 mg/kg, where the highest Mn content was detected in *J. rubens* while the lowest level was noted in *U. fasciata*, collected from Abu Qir Bay. Lower value of Mn 59.97 mg/kg was reported for *J. rubens* by Shams El-Din et al. [69] but a comparable level was observed for *U. fasciata* 16.01 mg/kg.

Zinc is found in the aquatic environment at low concentration and is necessary for some metabolism in living organisms at specific concentration [70]. It is a vital constituent of many enzymes such as carboxypeptidase, carbonic anhydrase, and several dehydrogenases. Zinc is not mutagenic and does not cause carcinogenic hazards to humans [71]. The concentration of Zn in the studied seaweeds fluctuated between 24.38 mg/kg in *C. sinuosa* and 93.47 mg/kg in *U. linza*. Significantly, higher value was observed by Ismail et al. [43] for the brown seaweed *S. wightii* (113.15 mg/kg) and lower concentration for the green seaweed *E. linza* (8.15 mg/kg). All studied species except *C. officinalis* and *U. linza* showed zinc concentration below the maximum permissible limit of zinc suggested for foods (50 mg/kg) [72]. The presence of high concentrations of Mn and Zn in seaweeds can be due to the fact that these elements are essential nutrients for metabolic functions of seaweeds [73].

Copper acts as a cofactor for various proteins and enzymes essential for maturation of cytoplasmic cuproproteins and assembly of enzymes in different cell organelles. Adequate intake of copper supplies protection against lead; however, higher intake results in increased lead absorption [74]. The excess amounts of Cu cause its involvement in the generation of highly reactive oxidative species having destructive effects in cells, especially DNA damage and oxidation of proteins and lipids [74]. The maximum value of Cu was observed in the green seaweed *U. linza* (19.27 mg/kg) and the lower value was detected in *J. rubens* (5.04 mg/kg). Ismail et al. [43] reported relatively higher level of Cu concentration 6.90 mg/kg for *J. rubens* and lower level for *E. linza* 2.80 mg/kg. The average concentration of Cu was found to be 12.70 ± 6.14 mg/kg. The Cu levels varied as *U. linza* > *U. fasciata (AQ)* > *U. fasciata (EH)* > *C. officinalis* > *C. sinuosa* > *J. rubens*. These values were found well within the range of 1.50–21.60 mg/kg reported for the marine algae from Brazil [75].

Cobalt plays a biologically important role as metal constituent of vitamin B12. Excessive exposure to Co causes various adverse health effects including cardiovascular, neurological, and endocrine deficits caused by the uptake of Co ions in the tissues and blood circulation [76]. Co concentration was in the range of 0.20 to 0.47 mg/kg for *U. fasciata* collected from Abu Qir Bay and *C. officinalis*, respectively. Al-Shwafi and Rushdi [77] indicated that the contents of Co in the green alga *Enteromorpha compressa* and the red alga *Hypnea cornuta*, collected from the Yemeni coastal waters of the Gulf of Aden, were 1.0 mg/kg and 0.14 mg/kg, respectively.

The permissible limit for chromium in raw herbal materials is 2.0 mg/kg [78]. All investigated seaweeds, except the red seaweeds *C. officinalis* and *J. rubens*, had Cr concentrations beyond the permissible limits defined by WHO. Cr concentration varied between 1.16 mg/kg (*C. officinalis*) and 8.02 mg/kg (*U. fasciata* collected from Abu Qir Bay) with the mean content of 3.99 ± 2.97 mg/kg. Sun et al. [79] reported lower value of 4.40 ± 0.0 mg/kg for *U. fasciata*. However, Chen et al. [80] determined relatively higher value for the red seaweed *Porphyra* samples, collected in coastal cities, China, than *C. officinalis* with the mean value of 1.64 ± 0.08 mg/kg and ranged from 0.31 to 7.05 mg/kg.

Nickel is extensively used in various industries so large amounts of nickel can enter the marine environment. At non-dangerous level, it may be useful for activation of some enzymes and taking part in important metabolic reactions. It is extremely carcinogenic and elevated levels of nickel cause gastrointestinal distress, shortness of breath, inhibition of oxidative enzyme activity, etc. [19]. The highest concentration of Ni was recorded in the green seaweed *U. fasciata* collected from Abu Qir Bay (5.51 mg/kg); however, the lowest level of Ni residue was observed in *J. rubens* (2.10 mg/kg). Shams El-Din et al. [69] reported lower concentration of Ni as 3.64 mg/kg in *U. fasciata* and higher Ni level of 11.97 mg/kg in *J. rubens* collected from the same area.

Lead is a potent toxicant with a widespread use which results in extensive contamination of the environment and causes critical health problems. Acute exposure leads to hypertension, renal dysfunction, abdominal pain, and arthritis [21, 74]. The seaweeds tested contained extremely lower levels of Pb compared to the permissible limit of 10 mg/kg defined by WHO [78] for a medicinal plant. Maximum amount of Pb 4.66 mg/kg was found in *C. officinalis* while the lowest level 0.89 mg/kg was recorded in *U. fasciata* obtained from Abu Qir Bay. Pb concentration was comparable in *U. fasciata* collected from the Eastern Harbor and *C. sinuosa.*

It has been reported that inhalation of cadmium fumes results in critical damage to respiratory system leading to shortness of breath [81]. Cd is a main causative factor for neurodegenerative disorders such as Alzheimer’s disease (AD) and Parkinson’s disease (PD) [81]. It also causes kidney damage, induction, and propagation of several types of cancers and tumors [81]. The highest Cd content 3.65 mg/kg was found in *J. rubens* while the lowest content 0.10 mg/kg was detected in *U. fasciata* obtained from Abu Qir Bay which is lower than the maximum level (0.50 mg/kg) set by French legislation for Cd in dry seaweeds [82]. It is also lower than the permissible limit (0.30 and 1.0 mg/kg) for raw herbal materials in Canada and China, respectively [78]. Ismail et al. [43] reported that *J. rubens* and *U. lactuca* had Cd content of 3.95 mg/kg and 0.10 mg/kg, respectively.

According to the WHO, the permissible limits of Pb, Cd, Cr, Cu, Ni, and Zn in the medicinal plant and food are 10, 1.0, 1, 50, 10, 15, and 50 mg/kg, respectively [64]. Furthermore, CEVA 2014 regulated the maximum tolerable level of 0.50 and 5.0 mg/kg for Cd and Pb, respectively in edible seaweeds for human consumption [83, 84]. Based on the permissible levels suggested by the WHO and CEVA, Pb and Ni in the studied seaweeds were found to be within the permissible limits, whereas Cr and Cu exceed the limit while Cd and Zn concentration was at the borderline.

Vanadium regulates the activity of key enzymes taking part in the phosphorylation and dephosphorylation of proteins, kinases, and phosphatases, involved in carbohydrate and lipid metabolism as well as in cell proliferation and differentiation [85]. However, the high amount of V in the environment and diet is hazardous to animals and humans [85]. The green alga *U. linza* recorded the highest vanadium content of 3.49 mg/kg followed by the brown alga *C. sinuosa* 3.13 mg/kg while *U. fasciata* collected from the Eastern Harbor exhibited the lowest level 0.58 mg/kg. Rubio et al. [86] found that brown seaweeds accumulate lower content of V (0.39 ± 0.55 mg/kg) which is opposite to our results while red seaweeds had higher level of V (6.68 ± 11.30 mg/kg).

Increased levels of arsenic (As) in foods comprise a food safety risk [21]. Intake of As can stimulate peripheral vascular and skin diseases, like hyperkerotosis [87]. In the present study, the concentration of As varied from 0.73 to 9.06 mg/kg. The higher concentration was observed in *U. linza* and the lower value was observed in *C. officinalis* which is below the allowable level (5.0 and 2.0 mg/kg) for raw herbal materials in Canada and China, respectively [78]. The average As concentration was 4.44 ± 3.05 mg/kg. Chen et al. [80] reported higher values of the mean concentration of total As 43.85 ± 1.42 mg/kg in the brown seaweed *Laminaria* samples and the red seaweed *Porphyra* samples 36.67 ± 0.53 mg/kg, collected from the same sampling site in China. Zhao et al. [88] found the total As of 9.84 ~ 16.9 mg/kg for *Porphyra*, collected from Xiamen, Fujian Province of China.

Selenium (Se) is an essential trace element which in small amounts is important for the development and health of humans and animals. It helps in the functions of the immune system and induces the production of antibodies [89]. It also contributes in various important metabolic interactions with some hazardous elements such as Hg and Cd [89]. The maximum amount of Se (1.09 mg/kg and 1.02 mg/kg) was recorded in both *C. sinuosa* and *C. officinalis*, respectively. Sun et al. [79] reported higher Se concentrations of 11.40 ± 0.10 mg/kg in *U. fasciata*, 4.90 ± 0.10 mg/kg in *Sargassum horneri*, 8.10 ± 0.10 mg/kg in *Pelvetia siliguosa*, 6.30 ± 0.10 mg/kg in *Laminaria japonica*, 27.5 ± 0.40 mg/kg in *Gracilaria lemaneiformis*, and 7.30 ± 0.10 mg/kg in *Gracilaria chouae* collected from different locations at Shen’ao Bay, China.

Molybdenum is an essential element for humans. It is a constituent of several enzymes which catalyze redox reactions [90]. The molybdenum cofactor is essential for the performance of three enzymes, namely sulfite oxidase, xanthine oxidase, and aldehyde oxidase. Molybdenum deficiency causes neurological symptoms and premature death [90]. In the studied seaweeds, the concentration of Mo varied from 0.05 to 0.30 where the highest content was observed in *C. sinuosa* followed by *J. rubens* (0.28 mg/kg), *U. fasciata* collected from Abu Qir Bay (0.25 mg/kg), and *U. linza* (0.20 mg/kg) while the lowest levels were detected in both *C. officinalis* (0.05 mg/kg) and *U. fasciata* (0.07 mg/kg) collected from the Eastern Harbor. Rubio et al. [86] reported lower values for brown (0.04 ± 0.01 mg/kg) and comparable values for red (0.16 ± 0.11 mg/kg) seaweeds.

It has been reported that the high content of barium can cause critical health problems. It affects the brain, the nervous system, and the liver [91]. In the present study, the highest content of Ba (34.93 mg/kg) was observed in *C. sinuosa* while the lowest level (6.07 mg/kg) was detected in *U. fasciata*, collected from the Eastern Harbor. In contrast, Rubio et al. [86] reported a much lower value (1.33 ± 0.38 mg/kg) for the brown seaweeds compared with *C. sinuosa.*

### Correlations

Tables 5, 6, and 7 show the statistically significant correlations between anti-inflammatory, anti-arthritic activities, the phytochemical content of hexane, dichloromethane, and ethanolic extracts vs. the heavy metal content in the investigated seaweeds, as well as the relationships between the metals themselves. For hexane extracts, significant positive correlations were found between the following pairs: carbohydrates vs. tannins (*r* = 0.9996) and Fe (*r* = 0.9997); proteins and Se (*r* = 0.9999); tannins and Fe (*r* = 0.9987); anti-inflammatory activity and Se (*r* = 0.9999). Significant correlations were also observed between the levels of individual metals (Table 5). For dichloromethane extracts, the correlations between phenols vs. anti-inflammatory activity (*r* = 0.9936), anti-arthritic activity (*r* = 0.9932), and Zn (*r* = 0.9299) are directly proportionate. Strong positive correlations were shown for tannins vs. Se (*r* = 0.8833) and Ba (*r* = 0.9855); anti-inflammatory activity vs. anti-arthritic activity (*r* = 0.9998) and Zn (*r* = 0.9567); and anti-arthritic activity vs. Zn (*r* = 0.9608). Strong negative correlations were also shown for Mn vs. Cu (− 0.8959) and Ni (*r* =  − 0.8852); Cu and Se (*r* =  − 0.8938); Cr and Pb (*r* =  − 0.9833); and Ni and Pb (*r* =  − 0.9512) (Table 6). For ethanolic extracts, significant positive correlations were demonstrated for proteins with tannins (*r* = 0.896) and anti-inflammatory activity and anti-arthritic activity (*r* = 0.9773). Strong positive and negative correlations were also obtained between the content of individual metals (Table 7).

**Table 5:** Statistically significant correlations between anti-inflammatory activity, phytochemical content of hexane extracts, and the heavy metal content in the investigated seaweeds

Correlations (*r*) between analyzed parameters, significant at *p* ≤ 0.05	
Anti-inflammatory activity	Se (*r* = 0.9999)
Carbohydrates	Tannins (*r* = 0.9996); Fe (*r* = 0.9997)
Proteins	Se (*r* = 0.9999)
Tannins	Fe (*r* = 0.9987)
Mn	Cr (*r* = − 1); Pb (*r* = 0.9998); Cd (*r* = 0.9987); Ba (*r* = 0.9978)
Cr	Pb (*r* = − 0.9997); Cd (*r* = − 0.9985); Ba (*r* = − 0.9975)
Pb	Cd (*r* = 0.9995); Ba (*r* = 0.9989)
Cd	Ba (*r* = 0.9999)
Mn	Se (*r* = 0.9999)

**Table 6:** Statistically significant correlations between anti-inflammatory, anti-arthritic activities, phytochemical content of dichloromethane extracts, and the heavy metal content in the investigated seaweeds

Correlations (*r*) between analyzed parameters, significant at *p* ≤ 0.05	
Anti-inflammatory activity	Anti-arthritic activity (*r* = 0.9998); Zn (*r* = 0.9567)
Anti-arthritic activity	Zn (*r* = 0.9608)
Phenols	Anti-inflammatory activity (*r* = 0.9936); anti-arthritic activity (*r* = 0.9932); Zn (*r* = 0.9299)
Tannins	Se (*r* = 0.8833); Ba (*r* = 0.9855)
Fe	V (*r* = 0.8854)
Mn	Cu (*r* = − 0.8959); Ni (*r* = − 0.8852); Cd (*r* = 0.9955)
Cu	Se (*r* = − 0.8938)
Cr	Ni (*r* = 0.8892); Pb (*r* = − 0.9833); As (*r* = 0.8825)
Ni	Pb (*r* = − 0.9512)

**Table 7:** Statistically significant correlations between anti-inflammatory, anti-arthritic activities, phytochemical content of ethanolic extracts, and the heavy metal content in the investigated seaweeds

Correlations (*r*) between analyzed parameters, significant at *p* ≤ 0.05	
Anti-inflammatory activity	Anti-arthritic activity (*r* = 0.9773)
Proteins	Tannins (*r* = 0.896)
Fe	Cr (*r* = 0.7183); Pb (*r* = − 0.6825); V (*r* = 0.9239); As (*r* = 0.8513)
Mn	Cu (*r* = − 0.9067); Cd (*r* = 0.7116)
Cu	Cr (*r* = 0.6426); Ni (*r* = 0.7883); Cd (*r* = − 0.9042); As (*r* = 0.6854)
Co	Pb (*r* = 0.7595); Se (*r* = 0.8314); Mo (*r* = − 0.6412); Ba (*r* = 0.6267)
Cr	Ni (*r* = 0.9036); Pb (*r* = − 0.9432); Cd (*r* = − 0.7704); V (*r* = 0.8798); As (*r* = 0.9429); Se (*r* = − 0.7248)
Ni	Pb (*r* = − 0.9002); Cd (*r* = − 0.9301); As (*r* = 0.8105); Se (*r* = − 0.7876)
Pb	Cd (*r* = 0.8284); V (*r* = − 0.7807); As (*r* = − 0.9269); Se (*r* = 0.9061)
Cd	As (*r* = − 0.7778); Se (*r* = 0.8467)
V	As (*r* = 0.8872); Mo (*r* = 0.6889)
As	Se (*r* = − 0.7842)

Tables 8, 9, and 10 demonstrate the significant correlations observed between antimicrobial activity, represented by AVG and AI, and other parameters in addition to the relationships between metals themselves. For hexane extracts, strong positive correlations were shown between AVG and AI (*r* = 1) and AI and Ni (*r* = 0.9994). For dichloromethane extracts, strong positive correlations were also shown for AVG vs. AI (*r* = 0.9996) and Se (*r* = 0.924) meaning that the higher the Se content, the higher antimicrobial activity obtained. Conversely, negative correlations with Cu (*r* =  − 0.9941) and Ni (*r* =  − 0.9029) were obtained. For ethanolic extracts, positive correlations were found between AVG vs. AI (*r* = 1) and tannins (*r* = 0.7279); AI and tannins (*r* = 0.7284); and proteins and tannins (*r* = 0.663). Moreover, strong correlations were demonstrated between the levels of individual metals in the tested seaweeds.

**Table 8:** Statistically significant correlations between antimicrobial activity of hexane extracts and the heavy metal content in the investigated seaweeds

Correlations (*r*) between analyzed parameters, significant at *p* ≤ 0.05	
AVG	AI (*r* = 1)
AI	Ni (*r* = 0.9994)
Fe	Zn (*r* = − 0.9991)
Co	Ba (*r* = 1)
V	Se (*r* = 0.9994)

**Table 9:** Statistically significant correlations between antimicrobial activity and phytochemical content of dichloromethane extracts and the heavy metal content in the investigated seaweeds

Correlations (*r*) between analyzed parameters, significant at *p* ≤ 0.05	
AVG	AI (*r* = 0.9996); Cu (*r* = − 0.9941); Ni (*r* = − 0.9029); Se (*r* = 0.924)
AI	Cu (*r* = − 0.9912); Ni (*r* = − 0.902); Se (*r* = 0.933)
Fe	V (*r* = 0.8854)
Mn	Cu (*r* = − 0.8959); Ni (*r* = − 0.8852); Cd (*r* = 0.9955)
Zn	Phenols (*r* = 0.9299)
Cu	Se (*r* = − 0.8938)
Cr	Ni (*r* = 0.8892); Pb (*r* = − 0.9833); As (*r* = 0.8825)
Ni	Pb (*r* = − 0.9512)
Se	Ba (*r* = 0.9158); tannins (*r* = 0.8833)
Ba	Tannins (*r* = 0.9855)

**Table 10:** Statistically significant correlations between antimicrobial activity and phytochemical content of ethanolic extracts and the heavy metal content in the investigated seaweeds

Correlations (*r*) between analyzed parameters, significant at *p* ≤ 0.05	
AVG	AI (*r* = 1); tannins (*r* = 0.7279)
AI	Tannins (*r* = 0.7284)
Proteins	Tannins (0.663)
Fe	Cr (*r* = 0.6214); Pb (*r* = − 0.6214); V (*r* = 0.9165); As (*r* = 0.8369); Mo (*r* = 0.627)
Mn	Zn (*r* = − 0.5899); Cu (*r* = − 0.8607); Ni (*r* = − 0.6616); Cd (*r* = 0.6897); Mo (*r* = 0.5889)
Zn	Cu (*r* = 0.6892)
Cu	Cr (*r* = 0.6384); Ni (*r* = 0.8005); Cd (*r* = − 0.7369); Se (*r* = − 0.7292); Ba (*r* = − 0.642)
Co	Pb (*r* = 0.755); Se (*r* = 0.6656)
Cr	Ni (*r* = 0.9041); Pb (*r* = − 0.9409); Cd (*r* = − 0.7527); V (*r* = 0.6824); As (*r* = 0.9049); Se (*r* = − 0.6944)
Ni	Pb (*r* = − 0.8838); Cd (*r* = − 0.8768); As (*r* = 0.741); Se (*r* = − 0.7938)
Pb	Cd (*r* = 0.8221); V (*r* = − 0.6449); As (*r* = − 0.9097); Se (*r* = 0.7894)
Cd	As (*r* = − 0.776); Se (*r* = 0.6723)
V	As (*r* = 0.8266); Mo (*r* = 0.7516)
As	Se (*r* = − 0.5819)
Se	Ba (*r* = 0.7197)

Generally, the strong positive correlations between the anti-inflammatory, anti-arthritic, and antimicrobial potential and some phytochemical contents in the seaweeds indicate that these compounds are closely linked to these activities. Similarly, the strong correlations between these activities and some trace metals.

### Estimated Daily Intake

Table 11 shows that *U. linza* had the highest values of estimated daily intake for Fe, Zn, Cu, As, and V (2.92E + 00, 3.03E − 01, 6.25E − 02, 2.94E − 02, and 1.13E − 02 mg/kg/day, respectively) while *C. sinuosa* possessed the highest values of EDI for Se, Mo, and Ba (3.54E − 03, 9.66E − 04, and 1.13E − 01 mg/kg/day, respectively). The values of estimated daily intake for Pb in all tested seaweeds were comparable to the levels set by European edible seaweeds (0.008–0.4 mg/kg/day) [82]. It ranged between 2.90E − 03 mg/kg/day in *U. fasciata* from Abu Qir Bay and 1.51E − 02 mg/kg/day in *C. officinalis* from the Eastern Harbor. The estimated daily intakes of Fe, Mn, Zn, Cu, Cr, Ni, V, Se, and Mo in all investigated seaweeds were lower than the recommended daily intakes for these elements [92, 93].

**Table 11:** Estimated daily intakes (EDI; mg/kg/day) for heavy metals of seaweeds collected from the Egyptian coast of Alexandria

Element	Green seaweeds			Red seaweeds		Brown seaweeds	Reference value*
	*U. fasciata (EH)*	*U. fasciata (AQ)*	*U. linza (AQ)*	*J. rubens (AQ)*	*C. officinalis (EH)*	*C. sinuosa (AQ)*	
Fe	4.76E − 01	1.31E + 00	2.92E + 00	1.35E + 00	2.56E − 01	1.85E + 00	8–18
Mn	7.96E − 02	5.74E − 02	6.85E − 02	2.77E − 01	6.23E − 02	1.64E − 01	1.8–2.3
Zn	1.32E − 01	1.40E − 01	3.03E − 01	1.00E − 01	2.37E − 01	7.91E − 02	8–11
Cu	5.39E − 02	5.46E − 02	6.25E − 02	1.63E − 02	4.25E − 02	1.73E − 02	0.9
Co	8.11E − 04	6.44E − 04	9.69E − 04	8.30E − 04	1.53E − 03	9.66E − 04	nd
Cr	8.51E − 03	2.60E − 02	2.43E − 02	6.50E − 03	3.76E − 03	8.54E − 03	(2.5–3.5)E − 02
Ni	1.23E − 02	1.79E − 02	1.44E − 02	6.80E − 03	7.90E − 03	8.21E − 03	1
Pb	8.35E − 03	2.90E − 03	3.71E − 03	1.08E − 02	1.51E − 02	9.66E − 03	3.57E − 03
Cd	1.49E − 03	3.22E − 04	1.05E − 03	1.18E − 02	9.45E − 03	4.94E − 03	1.00E − 03
V	1.86E − 03	8.78E − 03	1.13E − 02	5.59E − 03	2.61E − 03	1.01E − 02	1.8
As	1.09E − 02	2.14E − 02	2.94E − 02	6.80E − 03	2.38E − 03	1.55E − 02	nd
Se	9.73E − 05	1.62E − 04	9.73E − 05	1.74E − 03	3.29E − 03	3.54E − 03	5.50E − 02
Mo	2.27E − 04	8.05E − 04	6.46E − 04	9.08E − 04	1.62E − 04	9.66E − 04	4.50E − 02
Ba	1.97E − 02	2.50E − 02	4.00E − 02	3.86E − 02	4.06E − 02	1.13E − 01	nd

^*^Reference values are expressed as mg/kg/day for adult female and male (19–70) years and body weight 70 kg

*nd*, not determined

## Conclusions

The present work revealed that the tested seaweed extracts contain several bioactive compounds with anti-inflammatory and anti-arthritic potential which support the use of these extracts as natural agents for inflammatory disorders such as pains, infections, arthritis, and rheumatism. The tested extracts showed also varied antimicrobial activity against different human, animal, and plant pathogens which represent a promising scope to be used as eco-friendly biocontrol agents in the future. Further research should be undertaken on the separation, purification, and identification of the bioactive compounds from different seaweed species as well as trials to evaluate the efficiency of these purified compounds for medical uses in vivo. However, before being used in the pharmaceutical applications, the seaweeds have to be monitored for their heavy metal content to insure human safety. Therefore, there is a serious need for rapid assessment of these heavy metals for controlling the contaminant levels in the seaweeds.

## Supplementary Information

Below is the link to the electronic supplementary material.

Supplementary file1 (DOCX 75 KB)

## Data Availability

The datasets used and/or analyzed during the current study are available in this published paper and supplementary material.
