# Supplementary material for: Variation in Anti-inflammatory, Anti-arthritic, and Antimicrobial Activities of Different Extracts of Common Egyptian Seaweeds with an Emphasis on Their Phytochemical and Heavy Metal Contents
Source: Biol Trace Elem Res. 2022 Jun 4;201(4):2071–87. doi: 10.1007/s12011-022-03297-1 (PMC9931819; doi:10.1007/s12011-022-03297-1)
Supplement: Supplementary file 1 — Supplementary file1 (DOCX 75 KB) [file 12011_2022_3297_MOESM1_ESM.docx]

|  |  |
| --- | --- |
|  |  |
|  |  |
|  |  |
|  |  |
|  |  |

**Fig. 1S** Calibration curves of the studied metals
